# Genomic Profiling of Laryngeal Squamous Cell Carcinoma Reveals Novel Biomarkers for Precision Medicine

**DOI:** 10.3390/jpm16010002

**Published:** 2025-12-20

**Authors:** Beau Hsia, Gabriel A. Bitar, Nathan S. Tran, Katelin Keenehan, Pedro S. Bonilla, Saif Alshaka, Eli Oved, Peter T. Silberstein, Abubakar Tauseef, Vijay A. Patel, Aliasgher Khaku

**Affiliations:** 1 Creighton University School of Medicine, Phoenix, AZ 85012, USA; 2 Texas Tech University Health Sciences Center School of Medicine, Lubbock, TX 79430, USA; 3 Jacobs School of Medicine and Biomedical Sciences, University at Buffalo, Buffalo, NY 14203, USA; krkeeneh@buffalo.edu; 4 Michigan State University College of Human Medicine, Grand Rapids, MI 49503, USA; 5 Creighton University School of Medicine, Omaha, NE 68178, USA; 6 Division of Pediatric Otolaryngology, Rady Children’s Hospital, San Diego, CA 92123, USA; 7 Department of Otolaryngology-Head and Neck Surgery, University of California San Diego, San Diego, CA 92037, USA; 8 James A Haley VA Medical Center, Tampa, FL 33612, USA

**Keywords:** laryngeal squamous cell carcinoma, somatic mutation profiling, primary versus metastatic tumors, *TP53* and *KMT2D*

## Abstract

**Objective(s):** To characterize the somatic mutational landscape of laryngeal squamous cell carcinoma (LSCC) using AACR Project GENIE data to identify potential biomarkers for tumor progression and guide precision therapy. **Methods**: Clinical and genomic data from 135 LSCC samples (primary and metastatic) were analyzed from the AACR Project GENIE database. Mutations were compared by tumor site and gender using chi-squared and Mann–Whitney U tests; co-occurrence and mutual-exclusivity analyses were performed. **Results:** *TP53* mutations were most common (89.6%), followed by *KMT2D* (27.4%), *FAT1* (20.7%), and *NOTCH1* (20.7%). *CDK8* mutations were enriched in females (*p* = 0.011) and *ATP8B1* in males (*p* = 0.013). *DMD* mutations characterized primary tumors (*p* = 0.049), whereas *ATP8B1* and *SAMD9L* were linked to metastases (*p* < 0.001). The cohort was 85.9% male and 71.5% White; 59.2% of samples were primary and 39.2% recurrent/metastatic. Co-occurrence analysis identified distinct molecular subtypes. The identification of distinct molecular subtypes and gender-specific mutations, such as *CDK8* in females and *ATP8B1* in males, suggests potential avenues for tailored therapeutic interventions. **Conclusions:** LSCC exhibits marked genetic heterogeneity dominated by *TP53* alterations. *ATP8B1* and *SAMD9L* mutations may mark metastatic disease, and gender-specific mutations suggest avenues for personalized therapy. These insights support development of targeted strategies, including immunotherapies such as pembrolizumab in *TP53*-altered tumors. These insights into the genomic heterogeneity of LSCC lay the groundwork for developing targeted therapeutic strategies and patient stratification, ultimately advancing a personalized medicine approach to this disease.

## 1. Introduction

Larynx squamous cell carcinoma (LSCC) is a malignant epithelial tumor arising from the squamous cells lining the larynx, particularly affecting structures such as the glottis, supraglottis, and subglottis [1]. The United States alone was responsible for 12,650 new cases and 3880 deaths in 2024 [2]. This cancer exhibited notable disparities by race and sex, with men having a higher incidence than women (10,030 cases versus 2620) [2]. LSCC encompasses the majority of laryngeal cancers with the largest number of cases originating from the glottic region, followed by the supraglottic area [3]. Clinically, LSCC typically presents with progressive symptoms such as persistent hoarseness, dysphagia, throat pain, or respiratory obstruction, depending on the tumor’s location and size [4]. In contemporary oncology, the integration of Next-Generation Sequencing (NGS) and mutation diagnostics has become pivotal, transitioning treatment paradigms from histology-based to molecularly-driven precision medicine.

Several risk factors have been closely linked to LSCC, including prolonged tobacco use, excessive alcohol consumption, gastro-esophageal reflux, occupational exposures, as well as infection with high-risk human papillomavirus (HPV) strains, particularly HPV-16 and HPV-18 [5,6]. The etiological role of HPV in LSCC remains debated; while occasional coinfection has been reported, its contribution is likely limited compared to oropharyngeal SCC. Recently, there has been growing attention to how socioeconomic factors, including ethnicity, gender, age, type of insurance coverage, and geographic location, influence treatment outcomes and survival rates [7,8,9].

Approximately 90% of malignant neoplasms of the larynx are squamous cell carcinomas and can be graded as well, moderately, or poorly differentiated. Symptoms, treatment, and prognosis of LSCC vary depending on the subsite of onset. LSCC presents challenges due to its proximity to critical structures involved in breathing, speech and swallowing [6]. Supraglottic and subglottic cell carcinoma typically have worse outcomes compared to glottic SCC [10,11,12].

Treatment recommendations vary by tumor site and stage as well as patient factors. Early-stage LSCC (stages I and II) is typically treated with radiotherapy or endoscopic laser surgery in malignant neoplasms of the supraglottic and glottic larynx, which offer high rates of local control while preserving laryngeal function [13]. In contrast, advanced-stage LSCC (stages III and IV) frequently necessitates a multimodal approach, which involves total laryngectomy, chemotherapy, and radiotherapy [14,15,16]. While these aggressive treatments improve survival rates, they profoundly impact quality of life, often resulting in the loss of natural voice and changes to the airway [17]. Advanced laryngeal cancers have shifted from surgery as the gold standard treatment to primarily chemoradiotherapy, driven by clinical trials showing high rates of larynx preservation. However, concerns have arisen about reduced survival rates, and surgical salvage through total laryngectomy remains a viable option for post-radiotherapy recurrences confined to the larynx [18].

Despite advances in management, survival for advanced LSCC remains suboptimal, underscoring the need for improved molecular understanding. Prior studies such as TCGS have identified recurrent mutations in *TP53*, *NOTCH1*, *FAT1*, and *KMT2D*, but sex-specific and metastasis-specific landscapes remain poorly defined. This study aims to analyze the somatic mutational landscape of LSCC using AACR Project GENIE, focusing on distinctions between primary vs. metastatic tumors and male vs. female patients.

## 2. Materials and Methods

This study was exempt from the Creighton University institutional review board approval as the database is deidentified and publicly available. The American Association for Cancer Research (AACR) Project Genomics Evidence Neoplasia Information Exchange (GENIE)^®^ database was accessed using the cBioPortal (v16.1-public) online software [19] on 22 July 2024, with clinical data dating back to 2017. Genomic sequencing information from 19 international cancer centers is compiled in the AACR GENIE^®^ database. Only a select number of cancer types include therapeutic response along with clinical outcomes data, but treatment regimens were not recorded for LSCC. Additionally, each participating institution may use different pipelines from each other (and within the same institution). Participating institutions use either unbiased whole genomic/exome sequencing or targeted panels of up to 555 genes.

We queried all patients with head and neck tumors and a pathologic diagnosis of larynx squamous cell carcinoma. The dataset included genomic data (e.g., somatic mutations), histological subtype, as well as clinical characteristics (e.g., race and age). Specific copy number alterations and structural variants were excluded from this analysis. Tumor mutational burden was calculated based on the number of detected somatic mutations. Survival data was not available. Samples with missing data were excluded from the analysis to ensure the integrity of the results. Discrepancies in total numbers across different demographic categories (e.g., ethnicity versus sex) arise because not all participating institutions reported every data field for every patient; samples with missing specific data points were excluded from those specific sub-group analyses. Statistical analyses were conducted using R/R Studio (Version 4.5.2, R Foundation for Statistical Computing, Boston, MA, USA), with significance set at *p* < 0.05. Continuous variables were reported as means ± standard deviations (SD), and categorical variables were presented as frequencies and percentages. Differences between categorical variables were assessed using the chi-squared test. For comparisons of means between two groups, a two-sided T-test and nonparametric tests, such as the Mann–Whitney U test for non-normally distributed data, were applied. The Benjamini–Hochberg False Discovery Rate (FDR) correction was used to adjust for multiple comparisons.

## 3. Results

### 3.1. Larynx Squamous Cell Carcinoma Patients’ Demographics

The larynx squamous cell carcinoma patient demographics are described in detail in Table 1. 135 samples were taken from 130 adult patients. 116 (89.2%) were from males and 19 (14.6%) were from females. 93 (71.5%) patients were White, 11 (8.5%) were Asian, and 4 (3.1%) were Black. 77 (59.2%) samples were of the primary tumor and 51 (39.2%) were from metastasized tumors. Primary tumors had a mutation count ≤3 in 30 (62.5%) samples compared to 18 (37.5%) of metastasized samples. These results are outlined in Table 1.

### 3.2. Larynx Squamous Cell Carcinoma Top Somatic Mutations

**Figure 1:**
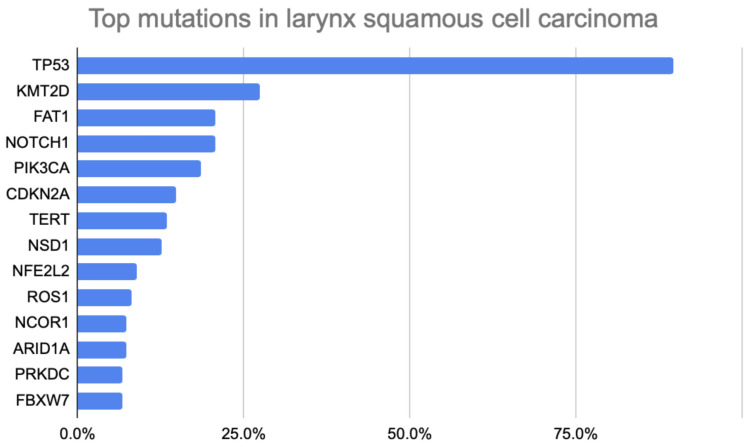
Bar Chart Illustrating Top Somatic Mutations in Larynx Squamous Cell Carcinoma Patients.

The top mutations detected in this larynx squamous cell carcinoma patient cohort are shown in Figure 1. Briefly, the most common mutations observed were in *TP53* (*n* = 121; 89.6%), *KMT2D* (*n* = 37; 27.4%), *FAT1* (*n* = 28; 20.7%), *NOTCH1* (*n* = 28; 20.7%), *PIK3CA* (*n* = 25; 18.5%), *CDKN2A* (*n* = 20; 14.8%), *TERT* (*n* = 18; 13.3%), *NSD1* (*n* = 17; 12.6%), *NFE2L2* (*n* = 12; 8.9%), *ROS1* (*n* = 11; 8.1%), *NCOR1* (*n* = 10; 7.4%), *ARID1A* (*n* = 10; 7.4%), *PRKDC* (*n* = 9; 6.7%), and *FBXW7* (*n* = 9; 6.7%). Specifically, co-occurrence was observed throughout mutated genes. Of the 25 patients with *PIK3CA* mutations, 11 (44.0%) had the *CDKN2A* mutation (*p* = 0.063).

### 3.3. Mutation Landscape in Key Genes

*TP53* mutations were diverse, with alterations such as *Y220C* and *R273H* identified. The allele frequency (AF), representing the proportion of a specific gene variant within a population, demonstrated significant variability among *TP53* mutations. Mutations such as *R248L* (AF = 0.69) and *G224C* (AF = 0.77) exhibited higher prevalence, indicating their occurrence in a larger proportion of the analyzed samples. Conversely, mutations such as *G226R* (AF = 0.04) were observed less frequently. Splice site mutations such as *X125*_splice (AF = 0.92) and nonsense mutations such as E204* (AF = 0.36) were also observed, emphasizing the range of *TP53* alterations in larynx squamous cell carcinoma.

### 3.4. Co-Occurrence and Mutual Exclusivity of Mutations

Of the 34 samples with either *KMT2D* or *PIK3CA* mutations, 9 (26.4%) had both mutations, although this did not represent statistically significant co-occurrence. Of the 17 samples that had the *NSD1* mutation, 4 (23.5%) had the *ROS1* mutation (*p* = 0.049). Co-*KMT2D* and *TP53* mutations were relatively mutually exclusive (*p* = 0.018) in larynx squamous cell carcinoma patients.

### 3.5. Gender Genetic Differences

When stratified by sex, female and male patients demonstrated significant enrichment of specific mutations. Mutations in *CDK8* (22.22% vs. 0%; *p* = 0.011) were exclusively seen in females. Conversely, mutations in *ATP8B1* (0% vs. 66.67%; *p* = 0.013) and *EPHB2* (0% vs. 50%; *p* = 0.024) were exclusively seen in males. Additional mutations such as *JAK3* (15.79% vs. 1.72%; *p* = 0.02), *MED12* (27.27% vs. 3.85%; *p* = 0.02), *KMT2B* (50% vs. 4.88%; *p* = 0.034), *GLI1* (18.18% vs. 1.30%; *p* = 0.04), and *FGF19* (44.44% vs. 13.85%; *p* = 0.045) occurred at higher frequencies in female patients when compared to male patients. These results are outlined in Table 2.

### 3.6. Primary vs. Metastatic Tumors

In this cohort, distinct mutational patterns were observed between primary and metastatic tumors. Among the 77 primary tumor samples, *DMD* (13.33%; *p* = 0.049) was identified exclusively in primary samples and absent in all metastatic samples. Additionally, mutations in *KMT2D* were also observed more frequently in primary samples when compared to metastatic samples (35.29% vs. 13.51%; *p* = 0.027). Within the 51 metastatic samples, mutations in *ATP8B1* (66.67%; *p* < 0.001), *FRK* (33.33%; *p* = 0.038), *NRIP1* (33.33%; *p* = 0.038), and *SAMD9L* (33.33%; *p* = 0.038) were identified exclusively in metastatic samples and absent in all primary samples.

In this cohort, among the 77 primary tumor samples, mutations were observed in *TP53* (*n* = 61; 79.2%), *KMT2D* (*n* = 18; 23.4%), *NOTCH1* (*n* = 14; 18.2%), *PIK3CA* (*n* = 15; 19.5%), *FAT1* (*n* = 7; 9.10%), *NSD1* (*n* = 6; 7.79%), *CDKN2A* (*n* = 9; 11.7%), *BRCA1* (*n* = 7; 9.10%), *ATM* (*n* = 7; 9.10%), *ROS1* (*n* = 6; 7.79%), and *ARID1A* (*n* = 7; 9.10%). In contrast, among the 51 metastatic tumor samples, mutations were observed in *TP53* (*n* = 38; 74.5%), *FAT1* (*n* = 12; 23.5%), *TERT* (*n* = 10; 19.6%), *PIK3CA* (*n* = 9; 17.6%), *NSD1* (*n* = 7; 13.7%), *CDKN2A* (*n* = 8; 15.7%), *NOTCH1* (*n* = 8; 15.7%), *HGF* (*n* = 5; 9.80%), *KMT2D* (*n* = 5; 9.80%), *PRKDC* (*n* = 4; 7.84%), and *NCOR1* (*n* = 2; 3.92%). These results are outlined in Table 3.

## 4. Discussion

In this study, we aimed to profile the somatic mutational landscape of laryngeal squamous cell carcinoma (LSCC) using the AACR GENIE database, with a particular focus on identifying actionable alterations that can inform personalized medicine strategies. LSCC is characterized by a high prevalence of TP53 mutations (89.6%), followed by *KMT2D* (27.4%), *FAT1* (20.7%), and *NOTCH1* (20.7%). These findings reaffirm prior work, including The Cancer Genome Atlas Program (TCGA) analyses, but extend them by examining metastatic samples and sex-based differences that have not been well-characterized in LSCC. Our analysis moves beyond cataloging mutations to interpreting their potential for guiding patient-specific therapies, a cornerstone of personalized medicine that seeks to tailor treatment to the individual molecular characteristics of a tumor.

### 4.1. Therapeutic Implications of Key Mutations and Pathways

The genomic alterations identified in this cohort underscore the potential for targeted therapeutic interventions in LSCC. Gender-specific variations were also noted, as *CDK8* mutations were exclusively present in females, whereas *ATP8B1* and *EPHB2* were exclusively seen in males. These results are intriguing but must be interpreted cautiously, given the small number of female patients. However, they open an important avenue for personalized medicine, suggesting that optimal treatment strategies may ultimately need to be stratified by sex. Validation in larger and more balanced cohorts will be essential to confirm these potential sex-linked genomic differences. Similarly, distinct mutational patterns emerged between primary and metastatic tumors, with *DMD* and *KMT2D* more prevalent in primary tumors, while *SAMD9L* and *ATP8B1* were found in metastases. Because samples were not paired, these differences should be viewed as hypothesis-generating rather than definitive evidence of metastatic drivers. Nevertheless, these findings highlight the critical need for genomic profiling of metastatic sites to guide personalized therapy in advanced disease, as the mutational landscape may evolve from the primary tumor.

Our findings align with previous research identifying *TP53* as the most frequently mutated gene in LSCC and a key driver of the poor prognosis and progression of tumors [20]. Disruptions in the gene impair DNA repair, apoptosis, and cell cycle regulation, contributing to aggressive tumor behavior and resistance to conventional therapies. While TP53 mutations have historically been challenging to target directly, they remain a critical biomarker for risk stratification. Developing strategies to counteract the effects of *TP53* loss is a major goal of personalized oncology. Compared to TCGA, which primarily included primary tumors, GENIE’s inclusion of recurrent/metastatic samples provides additional insight into disease progression. The Hedberg et al. study described mutational features of metastatic and recurrent HNSCC broadly [21]; however, our work isolates LSCC specifically, identifying *SAMD9L* and *ATP8B1* as candidate metastasis-associated mutations not previously reported in this tumor type. This distinction is vital for developing personalized approaches for patients with metastatic LSCC.

The gender differences observed in *CDK8*, *ATP8B1*, and *EPHB2* mutations are not well-established in the LSCC literature. Their identification here may suggest unexplored biological pathways influenced by sex, which is a growing area of interest in personalized medicine. Acknowledging these differences could lead to more effective, gender-specific treatment protocols. However, these findings also raise the possibility of false discovery given the limited sample size. Further validation in other datasets will be required to confirm these observations and explore their therapeutic relevance.

In a comprehensive review of parallel sequencing of head and neck squamous cell carcinomas, Nadal et al. confirm the role of *TP53* as the pivotal mutation in the profile of conventional head and neck squamous cell carcinomas. Additionally, they also found *TP53*, *FAT1*, *NOTCH1*, *KMT2C*, and *CDKN2A* to be the most frequently mutated genes in LSCC [22]. *KMT2D* mutations highlight the role of chromatin remodeling in LSCC. Prior research identifies *KMT2D* as a tumor suppressor, where loss-of-function mutations impair transcriptional regulation and promote oncogenesis. This finding aligns with studies demonstrating *KMT2D’s* role in early tumor development. In a systematic review of *KMT2D* involvement in the pathogenesis of head and neck cancer, Santos et al. found the gene’s involvement in cell cycle inhibitors leads to the accumulation of DNA damage and the tumor’s aggressive advancement [23]. Due to the high mutational frequency of the *KMT2D* gene in their review, they assert the strong association of the gene with tumor progression and its potential to serve as a biomarker for predicting immunotherapy response. Our findings of the mutual exclusivity of *KMT2D* and *TP53* mutations can be described in a study by Lee et al. in which they conclude that *KMT2D* can act as a coactivator of *TP53* as part of the ASCOM complex [24]. Loss of *KMT2D* has been shown to impair *TP53* target gene activation, mimicking the effects of *TP53* inactivation. Consequently, tumors with *KMT2D* mutations may not require *TP53* mutations to bypass tumor suppressor pathways. *Therefore*, a plausible explanation of the observed mutual exclusivity could be due to *KMT2D* tumors inactivating *TP53* function indirectly by disrupting chromatin remodeling and reducing accessibility to *TP53* target gene promoters. Conversely, *TP53* tumors could directly override *TP53* transcriptional activity, bypassing the need for functional *KMT2D*. These findings suggest that LSCC represents distinct molecular subtypes with unique vulnerabilities that should be explored further for targeted therapies. This molecular distinction is fundamental to personalized medicine, suggesting that patients with *KMT2D* mutations may respond to epigenetic therapies, while those with *TP53* mutations might require different strategies.

While most of our results align with existing studies, the unique identification of mutations such as *SAMD9L* and *ATP8B1* in metastatic tumors and gender-specific enrichment of *CDK8* and *EPHB2* are less commonly reported and may represent new areas for investigation in personalized therapeutics. *SAMD9L* mutations have been implicated in other cancers as contributors to tumor survival under stress, such as in gastric cancer and myelodysplastic syndrome; however, their role in LSCC metastasis is largely unexplored [25,26]. Similarly, *ATP8B1* mutations, associated with lipid metabolism, are rarely discussed in LSCC literature but have been proposed as a novel predictive biomarker in lung squamous cell carcinoma [27]. Although gender differences in cancer genetics are recognized in other malignancies, LSCC-specific studies have not reported these genetic patterns such as the mutations of *CDK8* in females and *ATP8B1* in males, warranting further investigation to determine if these could serve as sex-specific therapeutic targets or biomarkers.

Some studies report lower *TP53* mutation frequency in HPV-positive LSCC [22,28], as the E6 protein from the virus inactivates *TP53* functions directly, obviating the need for genetic mutations. On the other hand, HPV-negative tumors are dominated by alterations in tumor suppressor genes like *TP53* and *CDKN2A* as well as oncogenes *CCND1* and *MYC* [29]. This well-established dichotomy is a prime example of personalized medicine, where HPV status is already used to guide treatment and predict prognosis in head and neck cancers.

Our findings comparing primary and metastatic tumors add to the understanding of tumor progression from a personalized medicine perspective. In primary tumors, mutations in the *DMD* gene contribute significantly to tumor progression due to the loss of dystrophin expression. While altered dystrophin expression leads to the developmental onset of Duchenne muscular dystrophy, it also has been associated with downregulation across various malignancies and enrichment across multiple pathways within transcriptomes of primary tumors [30]. In a study by Jones et al., they describe the pathogenesis of *DMD* in several primary tumors including sarcomas, leukemias, lymphomas, and melanomas [31]. Similarly in mutations of the *KMT2D* gene, mutations drive the progression of tumor formation through distinct epigenetic mechanisms. By disrupting its role in chromatin remodeling and gene regulation, these mutations promote genetic instability, increasing susceptibility to further oncogenic changes and altering the tumor microenvironment by fostering immune evasion. Higher frequencies in primary mutations of the *KMT2D* gene have been highlighted in the literature including studies related to the mutational landscape of primary nasopharyngeal carcinomas and oropharyngeal cancers [32,33]. In metastatic tumors, the exclusive presence of mutations in *ATP8B1* and *FRK* in metastatic samples mirrors recent research on colorectal and breast cancers indicating that these genes play a role in tumor dissemination and metastatic colonization [34,35]. The potential of these mutations to serve as biomarkers for metastatic disease or as therapeutic targets aligns with ongoing research focused on identifying novel molecular markers for the early detection and management of metastasis [36,37,38]. This genomic divergence underscores the importance of serial biopsies and genomic monitoring in patients with advanced disease to tailor therapies to the evolving tumor landscape. However, it is important to note that these findings are exploratory given the lack of paired primary-metastatic samples in the database.

### 4.2. Current Experimental Therapies and Future Personalized Directions

Several emerging therapies align with the pathways indicated in this study, paving the way for a more personalized approach to LSCC treatment. For *TP53* mutations, intramural injections of *p*-53 and the use of cisplatin in radiation therapy are being explored in clinical trials for LSCC [39,40]. Cisplatin was also being explored in combination with cetuximab, docetaxel, and atezolizumab for targeting mutations in *PIK3CA* and high-risk HPV-negative cases [41]. The high frequency of mutations in chromatin-modifying genes like *KMT2D* suggests a potential vulnerability to epigenetic drugs, such as histone deacetylase (*HDAC*) inhibitors or *EZH2* inhibitors, which could be a promising personalized strategy for this patient subset. Some alternate pathways on therapeutics for LSCC include the use of nivolumab, carboplatin, and paclitaxel in the exploration of PDL1 expression, soy isoflavones in the expression of p16, COX-2, VEGF, IL6, p53, and Bcl-xL, and saracatinib on c-Src and downstream signaling molecules STAT3 and STAT5 [42,43,44,45,46,47,48]. Identifying which patients are most likely to benefit from these targeted agents based on their specific mutational profile is a key goal of personalized medicine.

Several limitations must be acknowledged. Participating GENIE institutions used heterogeneous sequencing platforms, ranging from targeted panels to whole-exome sequencing, introducing variability in mutation detection. Additionally, survival and treatment data were not available, limiting our ability to directly correlate genomic findings with clinical outcomes, a crucial step for translating this research into personalized clinical practice. Missing demographic and sample site data (e.g., race available for 108 of 130 patients) further complicates interpretation and explains why denominators vary across analyses. Lastly, the demographic skew towards males and white patients reduces the generalizability of findings across diverse populations.

Despite these limitations, this study provides significant insights into LSCC’s genetic landscape that are highly relevant to the field of personalized medicine. The prevalence of *TP53* and other mutations such as *KMT2D* and *NOTCH1* provides valuable insights into tumorigenesis, while distinctions between primary and metastatic tumors reveal potential markers for metastasis that could be used to guide treatment decisions. Furthermore, the mutational differences between genders offer new avenues for developing tailored therapeutic strategies. The study contributes to the growing body of evidence that genomic and epigenetic alterations in LSCC can inform targeted therapy development, moving beyond a one-size-fits-all approach. Future studies should integrate more diverse datasets, including transcriptomic and clinical outcomes, to substantiate these findings and maximize their translational impact.

In summary, this study contributes to the growing body of LSCC genomic research by highlighting both established drivers (*TP53*, *KMT2D*) and less characterized findings (*ATP8B1*, *SAMD9L*, *CDK8*). By framing these findings within the context of patient stratification and targeted therapy, this work underscores the immense potential of genomic profiling to usher in a new era of personalized medicine for patients with laryngeal cancer. These results should be interpreted as hypothesis-generating and provide a strong rationale for further studies integrating genomic, transcriptomic, and clinical outcome data to build a comprehensive molecular framework for individualized patient care.

## 5. Conclusions

This study provides a detailed characterization of the mutational landscape of laryngeal squamous cell carcinoma (LSCC), highlighting critical genetic alterations, particularly in *TP53*, *KMT2D*, *FAT1*, and *NOTCH1*. Notably, significant genetic heterogeneity exists between primary and metastatic tumors, with mutations in *ATP8B1* and *SAMD9L* prominently associated with metastasis. Additionally, gender-specific genetic variations, including *CDK8* mutations in females and *ATP8B1* mutations in males, suggest potential avenues for tailored therapeutic strategies. The identification of these molecular signatures contributes substantially to our understanding of LSCC tumorigenesis and progression, supporting the advancement of precision medicine approaches. Future research should integrate comprehensive genomic, transcriptomic, and clinical outcome data to further validate these findings and enhance therapeutic targeting in LSCC.

## Figures and Tables

**Table 1:** Larynx Squamous Cell Carcinoma Patient Demographics.

Patient Demographic	N (%)
**Ethnicity**	
Non-Spanish/non-Hispanic	98 (75.4)
Spanish/Hispanic	14 (10.8)
Unknown	2 (1.5)
**Race**	
White	93 (71.5)
Black	4 (3.1)
Asian	11 (8.5)
Other	5 (3.8)
Unknown	0 (0.0)
**Sex**	
Male	116 (85.9)
Female	19 (14.1)
**Age**	
Pediatric	0 (0.0)
Adult	135 (100)
**Sample Type**	
Primary	77 (59.2)
Metastasis	51 (39.2)
Not Collected	5 (3.8)

**Table 2:** Male vs. Female Demographics in Larynx Squamous Cell Carcinoma.

Patient Demographic	Male N (%)	Female N (%)
**Ethnicity**		
Non-Spanish/non-Hispanic	84 (72.4)	14 (73.7)
Spanish/Hispanic	13 (11.2)	1 (5.3)
Unknown	2 (1.7)	0 (0.0)
**Race**		
White	81 (69.8)	12 (63.2)
Black	2 (1.7)	2 (10.5)
Asian	10 (8.6)	1 (5.3)
Other	5 (4.3)	0 (0.0)
Unknown	0 (0.0)	0 (0.0)
**Age**		
Male	0 (0.0)	0 (0.0)
Female	116 (100.0)	19 (100.0)
**Sample Type**		
Primary	64 (55.2)	13 (68.4)
Metastasis	45 (38.8)	6 (31.6)
Not Collected	5 (4.3)	0 (0.0)

**Table 3:** Primary vs. Metastatic Larynx Squamous Cell Carcinoma Patient Demographics.

Patient Demographic	Primary N (%)	Metastasis N (%)
**Ethnicity**		
Non-Spanish/non-Hispanic	50 (64.9)	43 (84.3)
Spanish/Hispanic	12 (15.6)	2 (3.9)
Unknown	0 (0.0)	0 (0.0)
**Race**		
White	51 (66.2)	38 (74.5)
Black	1 (1.3)	2 (3.9)
Asian	8 (10.4)	3 (5.9)
Other	3 (3.9)	0 (0.0)
Unknown	0 (0.0)	0 (0.0)
**Sex**		
Male	64 (83.1)	45 (88.2)
Female	13 (16.9)	6 (11.8)
**Age**		
Pediatric	0 (0.0)	0 (0.0)
Adult	77 (100.0)	51 (100.0)

## Data Availability

The data presented in this study are available from the AACR GENIE Database at https://genie.cbioportal.org/ (accessed on 26 August 2025).
